# Optimization of *Arundo donax* Saccharification by (Hemi)cellulolytic Enzymes from *Pleurotus ostreatus*

**DOI:** 10.1155/2015/951871

**Published:** 2015-11-05

**Authors:** Rossana Liguori, Elena Ionata, Loredana Marcolongo, Luciana Porto de Souza Vandenberghe, Francesco La Cara, Vincenza Faraco

**Affiliations:** ^1^Department of Chemical Sciences, University of Naples “Federico II”, Complesso Universitario Monte S. Angelo, Via Cintia 4, 80126 Naples, Italy; ^2^Institute of Agro-Environment and Forest Biology, National Research Council (CNR), Via Pietro Castellino 111, 80131 Naples, Italy; ^3^Department of Bioprocess Engineering and Biotechnology, Federal University of Paraná, Coronel Francisco H. dos Santos Avenue 210, 81531-990 Curitiba, PR, Brazil

## Abstract

An enzymatic mixture of cellulases and xylanases was produced by *Pleurotus ostreatus* using microcrystalline cellulose as inducer, partially characterized and tested in the statistical analysis of *Arundo donax* bioconversion. The Plackett-Burman screening design was applied to identify the most significant parameters for the enzymatic hydrolysis of pretreated *A. donax*. As the most significant influence during the enzymatic hydrolysis of *A. donax* was exercised by the temperature (°C), pH, and time, the combined effect of these factors in the bioconversion by *P. ostreatus* cellulase and xylanase was analyzed by a 3^3^ factorial experimental design. It is worth noting that the best result of 480.10 mg of sugars/gds, obtained at 45°C, pH 3.5, and 96 hours of incubation, was significant also when compared with the results previously reached by process optimization with commercial enzymes.

## 1. Introduction

To solve the issues related to the petroleum-based energy and compounds, more attention has been focused on the use of lignocellulosic wastes as source for added-value bioproducts [1]. They show several advantages such as low cost and worldwide availability with an equal geographical distribution [2]. The major component of lignocellulosic materials is cellulose that is present in the cell wall within a matrix of hemicellulose and lignin bonded by cross-linkages. This complex structure requires mainly three steps for the conversion of lignocelluloses into added-valued bioproducts: (i) a pretreatment to remove lignin and expose the polysaccharides, (ii) hydrolysis of polysaccharides that can be performed enzymatically using an enzymatic cocktail composed of cellulases and hemicellulases, and (iii) fermentation of the sugars into the desired bioproducts.

The enzymatic hydrolysis represents the limiting step of the overall process due to the high costs of the employed enzymes cellulases, a group of enzymes comprising cellobiohydrolase (CBH),* endo*-1,4-*β*-D-glucanase (EG), and *β*-glucosidase (BG), and hemicellulases, including xylanase, xyloglucanase, mannanase, arabinase, galactanase, polygalacturonase, glucuronidase, acetyl xylan esterase, and other enzymes [3]. Extensive studies were reported in the last few decades in order to identify new more efficient enzymes [4–7] and to obtain a high yield of sugars with low enzymes dosage [8].

Filamentous fungi represent the major source of cellulases and hemicellulases and are able to produce large amounts of lignocellulosic enzymes in different growth conditions [9].

In this study, the basidiomycetous fungus* Pleurotus ostreatus* was employed as a source of (hemi)cellulolytic enzymes that were partially characterized and applied to the hydrolysis of the lignocellulosic biomass* Arundo donax*. This biomass was chosen since it can be cultivated on lands such as hilly areas that are considered not appropriate for the traditional cereal production because yield is low and because the traditional cropping system causes extreme vulnerability to soil erosion [10]. In these conditions, perennial biomass crops such as giant reed (*Arundo donax* L.) proved to reduce soil erosion and to increase potential gross income of farmers [11] with favourable environmental impacts [12]. This allows avoiding competition with the use of lands for food production.

In order to optimize the application of* P. ostreatus* (hemi)cellulolytic enzymes to* A. donax* hydrolysis, statistical analysis of biomass conversion by the investigated fungal enzymatic cocktail was performed. To identify the most significant parameters for the enzymatic hydrolysis, the Plackett-Burman screening design was applied and the combined effect of the most significant factors identified (temperature (°C), pH, and time) was analyzed by a 3^3^ factorial experimental design.

## 2. Materials and Methods

### 2.1. Microorganism

The strain* Pleurotus ostreatus* (Jacq.:Fr.) Kummer (type: Florida) (ATCC number MYA-2306) was maintained through periodic transfer at 4°C on solid medium containing 15 g/L agar and PDY [24 g/L potato dextrose (Difco, Detroit, Michigan, USA) and 5 g/L yeast extract (Difco)].

### 2.2. Preinoculum

Precultures were prepared by inoculating 500 mL of PDY broth in 1 L Erlenmeyer flask with six agar plugs (*⌀* = 11 mm) of* P. ostreatus* mycelium, from the edge of a 7-day-old agar culture, in a temperature-controlled incubator at 28°C on a rotary shaker at 120 rpm for six days. After homogenizing through sterile blender, the mycelia were washed with sterile distilled water three times under laminar flow cabinet. The washed mycelia were inoculated (10% v/v) in the medium A with the following composition: MgSO_4_·7H_2_O (0.3 g/L), FeSO_4_·7H_2_O (0.005 g/L), MnSO_4_·H_2_O (0.00156 g/L), ZnSO_4_·7H_2_O (0.0014 g/L), CaCl_2_ (0.3 g/L), CoCl_2_ (0.002 g/L), yeast extract (0.5 g/L), KH_2_PO_4_ (1.5 g/L), and pH 5.5.

### 2.3. Analysis of Inducers of* P. ostreatus* on Cellulase and Xylanase Activities Production

Preliminary experiments were carried out in 24-well plate flat bottom with Low Evaporation Lid (BD-Falcon, Franklin Lanes, New Jersey, USA) containing 1.5 mL of medium A and 10% v/v of homogenized mycelia of* P. ostreatus* in each well. The medium was supplemented with different carbon sources: xylan from beachwood (Sigma-Aldrich, St. Louis, MO, USA), carboxymethylcellulose (CMC), sodium salt medium viscosity (Sigma-Aldrich, St. Louis, MO, USA), 99% xylitol (Alfa Aesar, Parkridge Road, Ward Hill, MA, USA), D-(+)- 98% cellobiose (Alfa Aesar, Parkridge Road, Ward Hill, MA, USA), L(+)-Arabinose (Merck Millipore, Darmstadt, Germany), 98% L-(−)-Arabitol (Alfa Aesar, Parkridge Road, Ward Hill, MA, USA), microcrystalline cellulose (Alfa Aesar, Parkridge Road, Ward Hill, MA, USA), sophorose 0.6 mM (Sigma-Aldrich, St. Louis, MO, USA), D(+)-Xylose (Sigma-Aldrich, St. Louis, MO, USA), Arabinan (Megazyme), wheat arabinoxylan low viscosity (Megazyme), D-(+)Galactose (Sigma-Aldrich, St. Louis, MO, USA), and lactose (Carlo Erba, Milan, Italy), tested at final concentration of 1% (w/v), with the exception of the sophorose, tested at final concentration of 0.6 mM. The plates were incubated at 28°C on a rotary shaker at 250 rpm for 14 days. Samples were centrifuged at 13.000 rpm for 15 minutes and the supernatants were used for cellulase and xylanase assays.

The selected carbon sources were further investigated for their inductive effect at final concentration of 1% (w/v) in 1 L Erlenmeyer flask, containing 500 mL of medium and 10% v/v of homogenized* P. ostreatus* mycelia. The flasks were incubated at 28°C on a rotary shaker at 120 rpm for 35 days. Samples were collected for cellulase and xylanase assays as described above.

### 2.4. Enzymatic Activity Assays

#### 2.4.1. Xylanase Assay

Xylanase activity assay was performed according to Bailey et al. [13].

#### 2.4.2. Azo-CMCase Assay for Endo-1,4-*β*-Glucanase

Endo-1,4-*β*-Glucanase activity produced in liquid culture was assayed by using Azo-CMC (Megazyme, Ireland) as substrate, following supplier's instructions.

#### 2.4.3. Dinitrosalicylic Acid Assay for Endo-1,4-*β*-Glucanase

For assessing the optimum pH and temperature and thermal- and pH-resistance, endo-1,4-*β*-Glucanase activity was assayed towards CMC (Sigma-Aldrich, St. Louis, MO, USA) as substrate, following the DNS assay method reported by Ghose [14].

#### 2.4.4. *β*-Xylosidase, *β*-Glucosidase, and *α*-Arabinofuranosidase Assays

*β*-Xylosidase, *α*-arabinofuranosidase, and *β*-glucosidase activities were determined by using* p*-nitrophenyl-glycoside substrates as described in Marcolongo et al. [15]. All the enzymatic measurements were performed in triplicate.

### 2.5. Optimum Temperature and Thermoresistance

Supernatant of* P. ostreatus* was concentrated by ultrafiltration with a 10 kDa polyethersulfone membrane (Millipore Corporation, Bedford, MA, USA) and subjected to the determination of optimum temperature and thermoresistance of the xylanase and cellulase.

To assess the optimum temperature, the substrates birch-wood xylan (Sigma-Aldrich, St. Louis, MO, USA) and CMC (Sigma-Aldrich, St. Louis, MO, USA) used for the xylanase and cellulase activities assays, respectively, were dissolved in 50 mM Na citrate at pH 5.3 and the incubations in presence with the enzymatic preparation were performed at 30, 40, 50, 60, 70, and 80°C. The thermoresistance of the xylanase and cellulase activities was investigated by incubating the fungal culture supernatant in 50 mM Na citrate pH 5.3, at 30°C, 40°C, and 50°C.

The reported results correspond to mean values of the three independent experiments, each one performed in three replicates.

### 2.6. Optimum pH and pH Resistance

The optimum pH of* P. ostreatus* cellulase and xylanase was determined on the supernatant of fungal culture concentrated by ultrafiltration with a 10 kDa polyethersulfone membrane (Millipore Corporation, Bedford, MA, USA). The experiments were performed at 25°C using both McIlvaine buffer, with pH values between 3.0 and 9.0, and Na-citrate buffer, at pH ranging from 3.0 to 6.0, performing the cellulase and xylanase activities assays with the substrates CMC and birch-wood xylan, respectively, dissolved in the above-mentioned buffers.

The pH resistance of the cellulase and xylanase activities was analyzed by diluting the supernatant in McIlvaine buffer, with pH ranging from 3.0 to 9.0, and incubating at 25°C.

The reported results correspond to mean values of the three independent experiments each one performed in three replicates.

### 2.7. Enzymatic Hydrolysis

The enzymatic hydrolysis mixtures, set up according to the experimental designs, contained the pretreated* A. donax* biomass previously oven-dried at 50°C to a moisture content less than 10% (w/w) in a total volume of 2 mL consisting of 50 mM sodium citrate buffer plus the enzyme cocktail. The biomass hydrolysis was carried out with the following enzymatic preparations, whose units per grams of dry pretreated substrate are specified below in the following paragraph on Plackett-Burman (PB) design: cellulase and xylanase activities from* P. ostreatus* after 9 days of growth and the cellulase from* Trichoderma reesei* ATCC26921. The commercial enzymatic mix C (145 U/gds of cellobiase from* A. niger* and 8 U/gds of thermostable *β*-xylosidase) was also included in the reaction mix to obtain the complete carbohydrates hydrolysis into the respective monomers. The hydrolysis mixtures, supplemented with 40 g/mL tetracycline and 30 g/mL cycloheximide to prevent microbial contamination, were prepared in caped tubes and were incubated together with blanks (pretreated lignocellulosic material without enzyme cocktail) on a rotary thermoblock (Themomixer C, from Eppendorf) at 600 rpm. Different hydrolysis conditions were tested according to either “Plackett-Burman (PB) design” or “3^3^ factorial experimental designs.” Samples were withdrawn at different time intervals, chilled on ice, and centrifuged at 16.500 ×g for 30 min at 4°C.

The total released sugars were expressed as the amount (mg) of total soluble sugars liberated after hydrolysis per grams of pretreated biomass.

### 2.8. Determination of Sugar Content

The sugars contained in the cleared supernatants obtained from* A. donax*, pretreated and subjected to the two-step acid hydrolysis or the enzymatic hydrolysis as described above, were analyzed by a high-performance liquid chromatographic (HPLC) system (Dionex, Sunnyvale, CA, USA), equipped with an anionic exchange column (Carbopac PA-100) and a pulsed electrochemical detector. Glucose and xylose were separated with 16 mM sodium hydroxide at a flow rate of 0.25 mL/min and identified by the respective standards. Fucose was used as internal standard.

### 2.9. Experimental Design and Data Analysis

In order to elucidate the most significant conditions for the enzymatic hydrolysis of pretreated* A. donax* the Surface Response Methodology (SRM) was used. The Plackett-Burman (PB) factorial design, used to identify the critical parameters, and the 3^3^ factorial experimental design were obtained by the Statistica 12.0 software (Statsoft Inc., 2013). The regression coefficients, analysis of variance (ANOVA), and *p* and *F* values were used to estimate the statistical parameters employed by the same software. ANOVA table consists of calculations that provide information about levels of variability within a regression model and form a basis for tests of significance.

### 2.10. Plackett-Burman (PB) Design

A total of 11 (*N*) variables including temperature (°C), amount of biomass (%, w/v), pH, time (hours), concentration of cellulase and xylanase from* P. ostreatus* and of commercial cellulase from* Trichoderma reesei* ATCC26921 (U/gds), and 5 unassigned variables (dummy) were studied in 12 (*N* + 1) experiments. Each variable was examined at two levels, high and low, denoted by (+1) and (−1) signs, respectively (Table S1 in Supplementary Material available online at http://dx.doi.org/10.1155/2015/951871). Also 3 centre points, in which the medium level with the code (0) was considered for each parameter, were included in the PB matrix (Table 1). The main effect of each variable was determined using the following equation:

(1) $$\begin{array}{c} E_{xi}=\frac{\left(\sum M_{i+}-\sum M_{i-}\right)}{N}, \end{array}$$

where *E*_*xi*_ is the variable main effect and *M*_*i*+_ and *M*_*i*−_ are the sum of the responses in runs, in which the independent variable (*xi*) was present in high and low levels, respectively, while *N* is the half number of runs considered. If the main effect of the tested variable is positive, it means that the influence of the concerning variable is greater at the high level tested, and when it is negative, the influence of the given variable is greater at the low level.

### 2.11. 3^3^ Factorial Experimental Design

To investigate the effect of the most critical parameters, defined through the PB factorial design, as well as how their interactions affect the response variable, 3^3^ factorial experimental design was performed (Table S2). According to the 3^3^ full factorial design for the three variables, 27 experimental runs including 3 central points, totalizing 30 runs were executed (Table 2).

### 2.12. Validation of Developed Model

Based on the 3^3^ factorial experimental results, complementary experiments (in triplicate) were carried out to validate the developed model. The experiment performed incorporated the conditions (run number 18) that allowed obtaining the maximum amount of released sugars. After, the experimental responses obtained were compared to the theoretical responses calculated from the developed model. The difference between the experimental and theoretical responses was evaluated to test the reliability of the model to predict the yield of saccharification of* A. donax*.

## 3. Results and Discussion

### 3.1. Analysis of Inducers of Cellulase and Xylanase Production in* P. ostreatus*

A preliminary screening of the potential inducers of cellulase and xylanase activities production in* P. ostreatus* was firstly performed in 24-well plates monitoring the time course of the enzymes secretion for 14 days in the presence of 1% (w/v) CMC, microcrystalline cellulose, cellobiose, sophorose, xylan, xylose, xylitol, wheat arabinoxylan, galactose, lactose, and 0.6 mM sophorose. Many researches have been focused on induction of cellulase and xylanase production by different monosaccharides, disaccharides, and carbohydrates used as sole carbon source [16]. Generally, the final concentration of the sugar tested as inducer corresponds to the 1% (w/v), demonstrating that this amount gives an inductive effect on both cellulases [17, 18] and xylanases [19, 20]. As regards the sophorose, although in most cases it was used as carbon source at final concentration of 1% (w/v) [21–23], its inductive effect on cellulase activity was also reported at lower concentration [24].

This analysis revealed the effect of microcrystalline cellulose as inducer of cellulase and xylanase activities production (Figures S1 A-B), whilst no induction was evidenced in the tested conditions by the other compounds, although in literature they have been reported as strong inducers of (hemi)cellulases production by fungi, such as* Trichoderma reesei* [25],* Clostridium acetobutylicum* [26], and* Aspergillus niger* [27].

Based on the data obtained from the analyses in multiwell,* P. ostreatus* cultures in 1 L Erlenmeyer flasks containing 500 mL of medium with 1% microcrystalline cellulose were performed (Figures S2 A-B). The cellulase and xylanase activities reached a maximum value of 3.19 and 51.32 U/mL, respectively, after 9 days of fermentation. In many works, microcrystalline cellulose was reported as an inducer of cellulase and xylanase activities production in fungal strains, such as* Cerrena unicolor* VKM F-3196 [28],* Trichoderma viride* [29],* Streptomyces* sp. [30], and* Alternaria brassicae* [31]. When* C. unicolor* VKM F-3196 was grown in a medium containing microcrystalline cellulose, a production of 6.5 U/mL xylanase and 12.7 U/mL cellulase, at the fifth day of fermentation, was observed by Belova et al. [28]. Similar amounts of cellulase were produced by* Trichoderma viride*, cultivated on microcrystalline cellulose, reaching the highest value of 10.19 U/mg after 3 days of growth [29]. During submerged fermentation in presence of microcrystalline cellulose,* Streptomyces* sp. EC22 produced a maximum of cellulase (0.8 U/mL) and xylanase (2.4 U/mL) activity after 72 and 60 hours, respectively [30]. Ortega [31] showed the induction of extracellular cellulolytic enzymes for the fungus* Alternaria brassicae* grown in the presence of microcrystalline cellulose with a maximum of 1.95 and 10.90 U/mL for endoglucanase and xylanase activities, respectively, very low values in comparison with those hereby reported for the strain* P. ostreatus*.

### 3.2. Partial Characterization of* P. ostreatus* Cellulase and Xylanase Activities to Define the Enzymatic Properties Useful for Their Application

The analysis of the culture supernatant obtained after 9 days of growth of* P. ostreatus* revealed only very low *α*-arabinofuranosidase, *β*-glucosidase, and *β*-xylosidase activities whose concentrations were of 0.0082, 0.056, 0.0076 U/mL, respectively. These three enzymatic activities are needed to obtain the biomass bioconversion into fermentable sugars.

Moreover, the optimal temperature and pH, thermoresistance, and pH-stability of cellulase and xylanase activities produced by* P. ostreatus* were also evaluated to identify the conditions to be tested in the statistical analysis of* A. donax* bioconversion.

**Figure 1:**
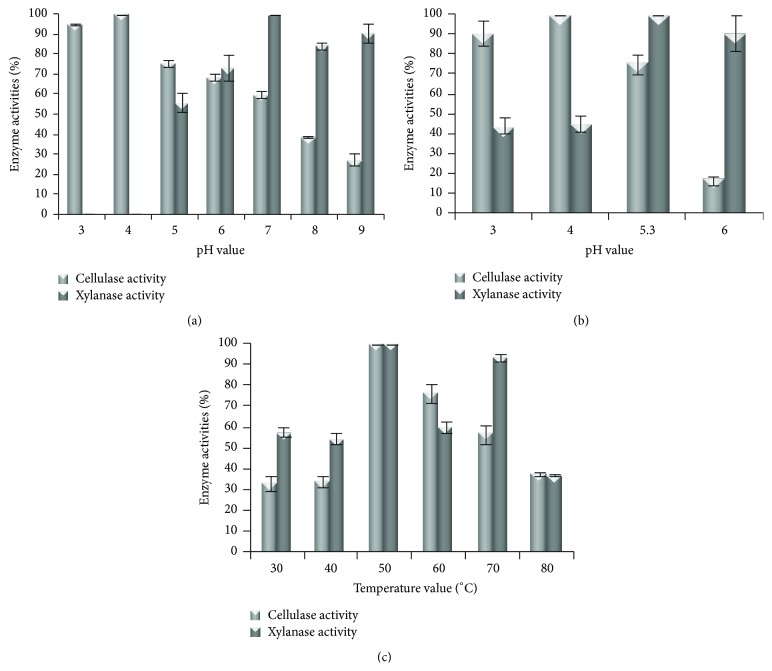
Effect of (a) pH in McIlvaine buffer, (b) pH in Na-citrate buffer, and (c) temperature on* Pleurotus ostreatus* cellulase and xylanase activities. The cellulase and xylanase activities were measured at pH ranging from 3.0 to 9.0 in McIlvaine buffer and from 3.0 to 6.0 in Na-citrate buffer and at the temperatures from 30 to 80°C.

In McIlvaine buffer, the optimal pH for the* P. ostreatus* cellulase activity was 4.0 (Figure 1(a)), similar to that reported for the three cellulases produced by* P. florida* (pH of 4.4) [32]. At least 60% of* P. ostreatus* cellulase activity was maintained in the range 3.0–7.0. Differently, the xylanase enzyme lost completely the activity at pH 3.0 and pH 4.0, showing an optimum at 7.0 (Figure 1(a)), a value close to the optimum of 6.0 reported for a xylanase produced by* P. ostreatus* [33].

In the Na-citrate buffer, the cellulase activity analyzed in our work showed an optimum at same pH value of 4.0, while a different optimal pH of 5.3 was observed for the xylanase (Figure 1(b)).

Both the* P. ostreatus* cellulase and xylanase activities showed an optimal temperature of 50°C (Figure 1(c)), which represents the condition mostly used for the enzymatic hydrolysis of lignocellulosic biomasses [15, 34]. The results were comparable to the optimum temperature of 45°C shown by the three cellulases produced by* P. florida* [32], while it was distant from that of xylanase produced by* P. ostreatus* (between 25 and 40°C) [33].

**Figure 2:**
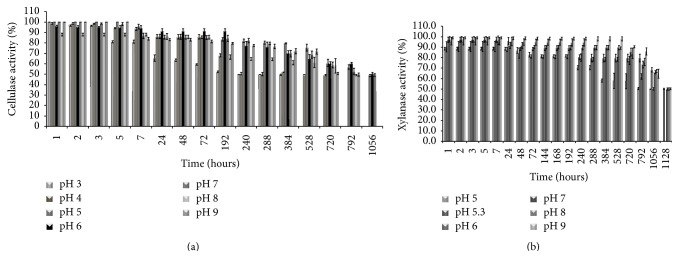
pH resistance of (a) cellulase and (b) xylanase activities of* Pleurotus ostreatus*. The pH resistance of the cellulase and xylanase activities was analyzed by diluting the supernatant in McIlvaine buffer, with pH ranging from 3.0 to 9.0, and incubating at 25°C. The percentage values reported in the graphs are referred to the initial enzymatic activities of 100%.

The* P. ostreatus* cellulase and xylanase activities showed very high stability in a broad range of pH values (Figures 2(a) and 2(b)). It is worth noting that the xylanase showed a more elevated stability than the other xylanase produced by* P. ostreatus* that loses 15 and 22% of activity at pH 6.5 and pH 9.0, respectively, after only 3 hours [33].

**Figure 3:**
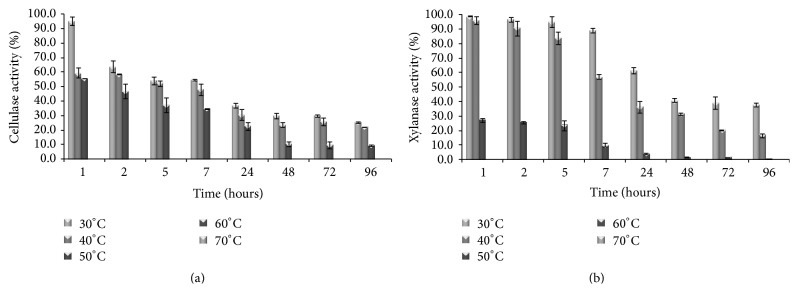
Thermoresistance of (a) cellulase and (b) xylanase activities of the strain* Pleurotus ostreatus*. The thermoresistance of the xylanase and cellulase activities was investigated by incubating the fungal culture supernatant in 50 mM Na citrate pH 5.3, at 30, 40, 50, 60, 70, and 80°C. The percentage values reported in the graphs are referred to the initial enzymatic activities of 100%.

Cellulase activity from* P. ostreatus* retained 50% of its value for at least 7 hours at 30°C and 40°C and 2 hours at 50°C and it immediately lost activity at temperature higher than 70°C (Figure 3(a)); other cellulases from* Pleurotus* sp., *β*-glucosidase, endoglucanase, and exoglucanase, showed a half-life of 15 minutes at 72, 66, and 58°C, respectively [32]. Xylanase activity from* P. ostreatus* retained 50% of its activity for at least 2 days at 30°C and 7 hours at 40°C (Figure 3(a)); like the other xylanase from* P. ostreatus* [33], it lost the activity at temperatures higher than 50°C.

### 3.3. Screening of Parameters Affecting the Enzymatic Hydrolysis by PB Design

Statistical analysis of* Arundo donax* bioconversion by cellulases and xylanases produced by* Pleurotus ostreatus* after 9 days of growth in the presence of microcrystalline cellulose was performed. Carbohydrate compositions of the untreated and pretreated [35] giant reed (*Arundo donax*) are reported in Table 3.

The effect of the six parameters, temperature (°C), biomass (%, w/v), pH, cellulase from* P. ostreatus* (U/gds), commercial cellulase from* Trichoderma reesei* ATCC26921 (U/gds), and incubation time (hours), on the sugars released during the enzymatic hydrolysis by* P. ostreatus* extracellular cellulase and xylanase was analyzed through the PB screening design.

Since in the enzymatic cocktail produced by* Pleurotus ostreatus* after 9 days of growth the cellulase activities were lower than the xylanase one, the only cellulase activity level was chosen as parameter in the statistical analysis experiments.

Moreover, the addition of the commercial enzymatic mix C (145 U/gds of cellobiase from* A. niger* and 8 U/gds of thermostable *β*-xylosidase) in the hydrolysis reaction was necessary to obtain the complete carbohydrates hydrolysis into the respective monomers, since the *β*-glucosidase and *β*-xylosidase activities were not detected in the supernatant of the strain* P. ostreatus*.

**Figure 4:**
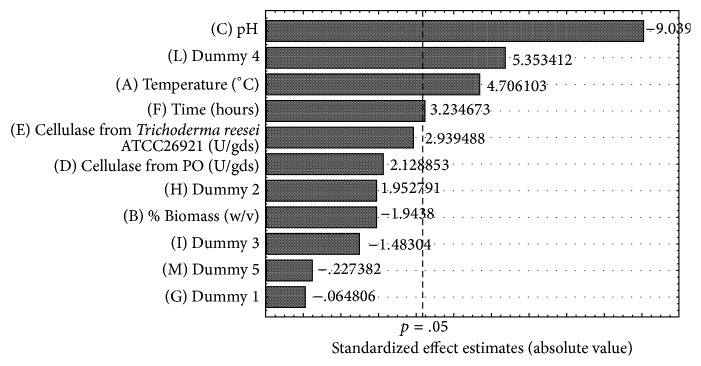
Pareto Chart of the Plackett-Burman design of sugars released during the enzymatic hydrolysis of* Arundo donax* (*R* ^2^ = 0.98 and *R*_adj._^2^ = 0.92), showing the significant factors at 95% of confidence level (*p* < 0.05). Significant factors were pH (*p* = 0.002859), temperature (*p* = 0.018157), and time (*p* = 0.048045) with an effect of −155.83, +104.69, and +71.96, respectively.

In Table S1 the lowest (−1) and the highest (+1) values tested for each factor were reported. The results of the analysis were reported in Table 1, ANOVA data were reported in Table S3, and the Pareto Chart, showing the standardized effects of the analyzed factors, was presented in Figure 4.

As reported in the ANOVA Table (Table S3), the most significant influence on the released sugars during the enzymatic hydrolysis of* A. donax* was exercised by the temperature (°C), pH, and time. In Figure 4, the Pareto Chart (*R* ^2^ = 0.98 and *R*_adj._^2^ = 0.92) shows that the three significant factors at 95% of confidence level (*p* < 0.05) are the pH representing the most important factor for the released sugars (*p* = 0.002859) and exercising a strong negative effect of −155.83, followed by the temperature (*p* = 0.018157) and the time (*p* = 0.048045) with a positive effect of +104.69 and +71.96, respectively.

Within the tested experimental values, the other three variables, dosage of cellulase from* T. reesei* ATCC 26921, dosage of cellulase from* P. ostreatus*, and % (w/v) of biomass, did not show significant effect at 95% of confidence level on the hydrolysis of saccharification of* A. donax*. The dosage of cellulase from* Trichoderma reesei* ATCC 26921 and that from* P. ostreatus* affected the saccharification of* A. donax* at 94 and 88% confidence levels, respectively.

Moreover, the PB analysis showed a strong influence of the dummy 4, one of the unassigned considered variables. As explained by Stowe and Mayer [36] (1966), this may be due to an experimental error or a possible interaction between two factors not revealed by the PB analysis but by the 3^3^ full factorial analysis.

### 3.4. Analysis of Combined Effect of pH, Temperature, and Time on the Bioconversion of* Arundo donax* through the 3^3^ Factorial Experimental Design and Validation of Generated Model

Since the PB analysis showed that the pH, temperature (°C), and time (hours) were the most significant factors on the sugars released during the enzymatic hydrolysis of* A. donax*, a 3^3^ full factorial experimental design with 30 runs was performed to analyze the combined effect of them.

As regards the other factors, % (w/v) of biomass, U/gds of cellulase from* P. ostreatus*, and U/gds of commercial cellulase from* T. reesei* ATCC26921, since they had no effect on the released sugars, the experiments were performed using their level 0 tested in the PB analysis, in order to reduce the utilization of both commercial cellulase and enzymes from* P. ostreatus*, with economical advantage. Also, for the % (w/v) of biomass, it was chosen to adopt the level 0 (5% w/v) since previous experiments had shown better results with this biomass percentage [37].

**Figure 5:**
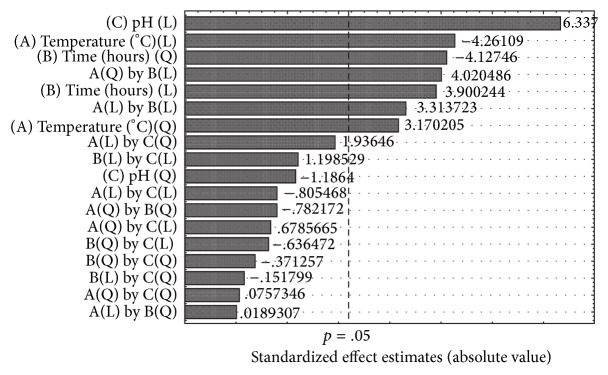
Pareto Chart of the 3^3^ full factorial design of sugars released during the enzymatic hydrolysis of* Arundo donax* (*R* ^2^ = 0.93; *R*_adj._^2^ = 0.80), showing the significant factors at 95% of confidence level (*p* < 0.05). Significant factors were pH (*p* = 0.000183), temperature (*p* = 0.000921), and time (*p* = 0.000537) with an effect of −105.85, −71.20, and +65.13, respectively. Strong positive impact on the bioconversion process was exercised also by the interaction between the temperature and the time.

In Table S2, the lowest (−1) and the highest (+1) values tested for each factor in the 3^3^ full factorial experimental design were reported. The results of the analysis were reported in Table 2, ANOVA data were reported in Table S4, and the Pareto Chart, showing the standardized effects of the analyzed factors, was presented in Figure 5.

The best result of 480.10 mg of sugars/gds was given by the following combination of factors (run number 18): 45°C, pH 3.5, and 96 hours of incubation (Figure 5).

The ANOVA Table (Table S4) showed that all the tested factors (temperature, pH, and time) exercised a strong influence on the sugars released during the enzymatic hydrolysis of* A. donax*. The 95% confidence interval and a coefficient of determination *R* ^2^ of 0.93, (*R*_adj._^2^ of 0.80) indicated that the model was statistically significant. As shown in the Pareto Chart (Figure 5), the pH was the most significant factor for the released sugars (*p* = 0.000183), in agreement with the results obtained by PB analysis, and it exercised a strong negative effect of −105.85; it is followed by the temperature (*p* = 0.000921) and the time (*p* = 0.000537) with a negative effect of −71.20 and a positive effect of +65.13, respectively.

A relevant positive impact on the bioconversion process was exercised by the interaction between temperature and time. This strong interaction could explain the influence of the dummy 4 revealed by the PB analysis, excluding the hypothesis of an experimental error [36].

**Figure 6:**
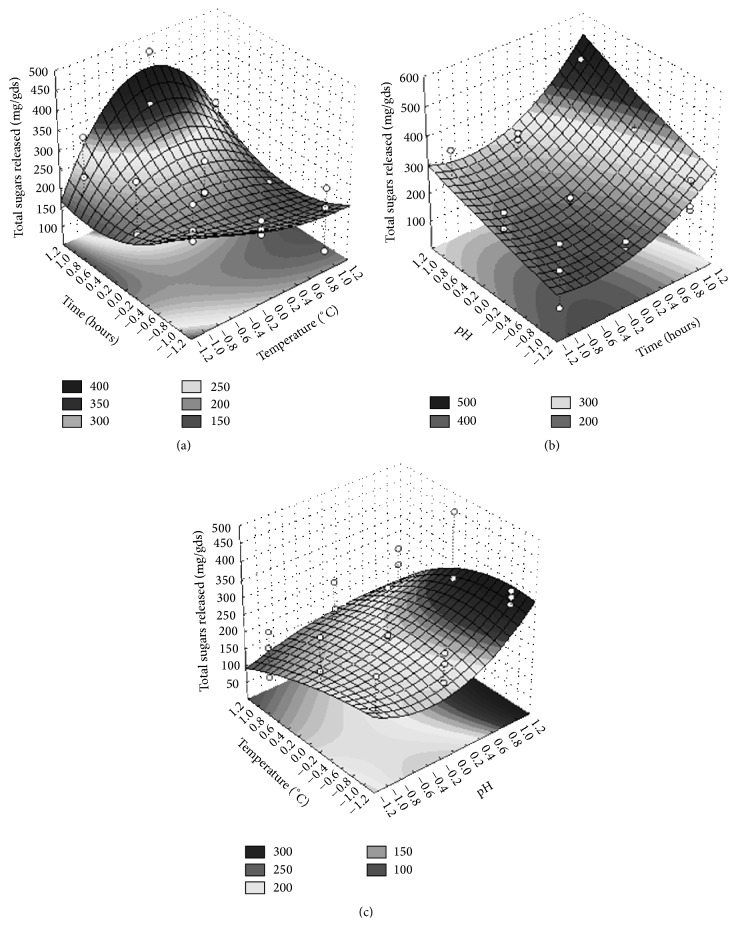
Surface Response showing the optimum region of each variable (central point) for the highest sugars released (mg/gds) during the hydrolysis of* Arundo donax*.

The 3D response surface was obtained by plotting the response values (mg glucose/g dry substrate) on the *Z* axis against (1) the variables temperature and time, keeping the pH constant at its level 0 (Figure 6(a)), (2) the variables pH and time, keeping the temperature constant at its level 0 (Figure 6(b)), and (3) the variables temperature and pH, keeping the time constant at its level 0 (Figure 6(c)). The Surface Response is demonstrated by

(2) Y=206.25−12.49∗A−30.81∗A2+78.27∗B+79.39∗B2+53.44∗C+22.14∗C2+33.90∗AB−8.25∗AC−0.33∗AB2−34.32∗AC2−71.23∗A2B−12.04∗A2C−22.22∗A2B2+2.13∗A2C2+12.24∗BC+2.67∗BC2+11.26∗B2C−10.56∗B2C2,

where *Y* indicates the released sugars concentration (mg/g dry substrate), *A* is the temperature (°C), *B* is the time (hours), and *C* is the pH.

The saccharification ratio increased with increase in time of incubation, keeping constant the pH at 3.5. Increase of temperature in the range from 35 to 45°C resulted in an improvement of the sugars yield. This trend, due to the positive interaction between temperature and time, gave more pronounced effect at longer incubation times with the maximum sugar recovery at 96 h. At this time a further temperature increase generated a reduction of saccharification yield (Figure 6).

The experimental results (average) did not differ significantly with the theoretical values obtained by the generated model (Table S5). Based on the determination coefficient (*R* ^2^ = 0.93 and *R*_adj._^2^ = 0.80), more than 80% of the results are represented by the model. The plotting between the experimental total reducing sugars and the predicted total reducing sugars was reported in Figure S3.

The conditions under which the maximum value of the sugars released was achieved were also verified by carrying out the experiments corresponding of run 18, whose results were in close agreement with the model prediction (455 mg/gds).

In general, the sugars released by the bioconversion of the pretreated* A. donax* reported in this study were not so far from those reported until now in literature, also in comparison with the results obtained using commercial enzymes. Several sources of enzymes were reported in the bioconversion of lignocellulosic biomasses and the different sugars yield obtained, which are summarized in Table 4, were influenced by the type of pretreated lignocellulosic materials, the enzymatic cocktail, and the operative conditions adopted in the process.

It is worth noting that the bioconversion of the pretreated* A. donax* reported in this study released similar amounts of glucose (229.30 mg/gds) and xylose (250.80 mg/gds) to those previously obtained by using commercial enzymes cocktail, containing 60 FPU/gds of cellulases and 64 pNPGU/gds of *β*-glucosidases, in the bioconversion of the same biomass, which released 264.0 mg glucose/gds and 217.0 mg xylose/gds [38] (Table 4). Rana et al. [39] showed that the enzymatic bioconversion of biological pretreated* Parthenium sp.* with the Accellerase 1500 (Novozymes) allowed obtaining 485.64 mg of total sugars per gram of dry substrate (Table 4), comparable to that (480.10 mg/gds) obtained in this study.

In some cases, the amount of sugars obtained using crude enzymes from fungi in lignocellulose bioconversion, that is, from* Aspergillus candidus* in saccharification of aqueous ammonia treated corn cob or from* Aspergillus foetidus* MTCC 4898 in saccharification of NaOH pretreated agricultural residues like wheat straw, rice straw, and corncobs [40, 41], was lower than that obtained in our study (Table 4).

Many works concerning the statistical optimization of pretreated lignocellulosic materials bioconversion, applying either commercial enzymes or enzymes from different microorganisms, were so far reported in literature. Lower sugars yield (293 mg/g substrate) than those reported in this work were obtained from the saccharification of* Populus balsamifera*, under optimized conditions, 65 FPU of cellulases from* Agaricus arvensis*/g substrate, 10% of the substrate, and a temperature of 40°C [42] (Table 4); while higher sugars yield, comparable to those obtained by using* P. ostreatus* enzymes, was reported by Phuengjayaem et al. [43].

It is noteworthy that the sugars' yield hereby reported with* P. ostreatus* enzymes was significant also when compared with that reached by process optimization with commercial enzymes. In fact, maximum reducing sugar yield of 266.0 mg/g was found, under optimized conditions, for the enzymatic hydrolysis of apple pomace by a commercial cocktail (43.0 U/g of Celluclast 1.5 L, 183.0 U/g of Pectinex 3XL, and 41.0 U/g of Novozyme 188), as reported by Parmar and Rupasinghe [44] (Table 4). Ferreira et al. [45], through response surface methodology, obtained a maximum reducing sugar yield of 313.0 mg/g for* Cistus ladanifer* and 418.0 mg/g for* Cytisus striatus* by using 60.0 FPU/g of cellulase complex NS50013 and *β*-glucosidase NS50010 (Table 4).

Moreover, at the best assessed conditions, the enzymatic cocktail produced by* P. ostreatus* allowed obtaining, at the same time, both glucose and xylose at similar yields. Differently, Ruangmee and Sangwichien [46] showed that at the optimal conditions for glucose yield (552.9 mg/g substrate), the xylose yield (74.0 mg/g substrate) was much lower.

Furthermore, it is worthy to note that an extensive literature search reveals that (hemi)cellulolytic enzymes produced by* P. ostreatus* represent a good candidate as biocatalyst for the enzymatic saccharification of* A. donax*, since they give a sugar yield that was only in few cases a little bit lower [47, 49] or comparable [40, 43] whilst, in some other cases, it results higher than that obtained by using enzymes from different sources [47–49], including the commercial ones [50–52].

## 4. Conclusions

It was demonstrated that microcrystalline cellulose is able to induce the* Pleurotus ostreatus* production of a mixture of cellulases and xylanases which were shown to be able to hydrolyze the pretreated* Arundo donax*. A statistical analysis of bioconversion based on this mixture led to the best results of 480.10 mg of sugars/gds at 45°C, pH 3.5, and 96 hours of incubation. In these conditions, a significant sugar yield was obtained also in comparison with results previously reported in literature. Interestingly, both glucose and xylose at similar yields were achieved at the same time.

## Supplementary Material

In the supplementary materials are provided: Table S1 indicating the levels decoded of the tested parameters in the Plackett-Burman Screening Design; Table S2 in which are reported the independent factors (Temperature, Time and pH) and their respective coded and decoded levels used for the 33 experimental design of the optimization of the enzymatic hydrolysis of *Arundo donax*; Table S3 showing the Analysis of Variance (Anova) of Plackett-Burman Screening Design for the enzymatic hydrolysis of *Arundo donax*; Table S4 showing the Anova of the 33 factorial experimental design for the optimization of enzymatic hydrolysis parameters and Table S5 in which is reported the comparison of observed and predicted values of the 33 factorial experimental design of sugars released (mg/gds) during the enzymatic hydrolysis of *Arundo donax*. Moreover, they provided: Figure S1 showing the inducer effect of 1% microcrystalline cellulose on cellulase and xylanase activities production by *Pleurotus ostreatus* in 24-Multiwell; Figure S2 showing the inductive effect of 1% microcrystalline cellulose on cellulase and xylanase activities production by *Pleurotus ostreatus* in 1 L Erlenmeyer flask and Figure S3 in which is reported the correlation between observed and predicted values of the total sugars released by enzymatic hydrolysis of *Arundo donax*.

## Figures and Tables

**Table 1:** Plackett-Burman 11/12 screening design: two levels, 6 factors, 3 central points, and 5 dummy factors (D1, D2, D3, D4, and D5).

Run	Temperature (°C)	% biomass (w/v)	pH	Cellulase from PO (U/gds)^a^	Cellulase from *Trichoderma reesei* ATCC26921 (U/gds)	Time (hours)	Dummy 1	Dummy 2	Dummy 3	Dummy 4	Dummy 5	Total sugars released (mg/gds)
1	45	7	3	10	1.8	24	1	1	1	−1	1	179.15
2	45	3	6	30	1.8	24	−1	1	1	1	−1	509.95
3	25	7	6	10	5.4	24	−1	−1	1	1	1	173.70
4	45	7	3	30	1.8	72	−1	−1	−1	1	1	408.55
5	45	3	6	30	5.4	24	1	−1	−1	−1	1	439.30
6	45	7	6	10	5.4	72	−1	1	−1	−1	−1	312.75
7	25	7	6	30	1.8	72	1	−1	1	−1	−1	112.15
8	25	7	3	30	5.4	24	1	1	−1	1	−1	344.35
9	25	3	3	30	5.4	72	−1	1	1	−1	1	461.70
10	45	3	3	10	5.4	72	1	−1	1	1	−1	598.30
11	25	3	6	10	1.8	72	1	1	−1	1	1	456.35
12	25	3	3	10	1.8	24	−1	−1	−1	−1	−1	271.60
12C	35	5	4.5	20	3.6	48	0	0	0	0	0	387.40
14C	35	5	4.5	20	3.6	48	0	0	0	0	0	419.30
15C	35	5	4.5	20	3.6	48	0	0	0	0	0	355.50

^a^Due to the copresence of two (hemi)cellulolytic activities in the *P*. *ostreatus* supernatant, when 10, 20, and 30 U/gds of cellulase are utilized in the different experimental runs, the xylanase concentrations in the enzymatic hydrolytic mixtures are 200, 400, and 600 U/gds, respectively.

**Table 2:** 3^3^ experimental screening design: three levels, 3 factors, and 3 central points.

Run	*T* (°C)	Time (hours)	pH	Total sugars released (mg/gds)
1	35	48	2.5	289.90
2	35	48	3	264.40
3	35	48	3.5	353.30
4	35	72	2.5	184.60
5	35	72	3	180.60
6	35	72	3.5	315.80
7	35	96	2.5	187.50
8	35	96	3	233.80
9	35	96	3.5	335.90
10	45	48	2.5	196.90
11	45	48	3	209.10
12	45	48	3.5	233.90
12	45	72	2.5	150.30
14	45	72	3	207.40
15	45	72	3.5	291.70
16	45	96	2.5	292.80
17	45	96	3	347.40
18	45	96	3.5	480.10
19	55	48	2.5	69.40
20	55	48	3	185.0
21	55	48	3.5	235.60
22	55	72	2.5	156.70
23	55	72	3	155.50
24	55	72	3.5	156.60
25	55	96	2.5	203.18
26	55	96	3	262.0
27	55	96	3.5	281.0
28C	45	72	3	210.9
29C	45	72	3	211.0
30C	45	72	3	210.5

**Table 3:** Macromolecular composition of untreated and pretreated *Arundo donax*.

	Carbohydrate composition	
	(% total dry weight)	
	Untreated	Pretreated
Glucan	26.3 ± 1.6	38.2 ± 1.2
Xylan	24.1 ± 1.2	5.7 ± 0.9
Klason lignin	9.8 ± 0.4	36.1 ± 0.6

**Table 4:** Comparison of enzymatic hydrolysis of different pretreated lignocellulosic biomasses by different enzyme sources and the obtained sugars released (mg/gds).

Biomass	Pretreatment	Glucose release (mg/gds)	Xylose release (mg/gds)	Enzyme used	References
Narrow-leaf cattail	Alkali pretreatment	552.9	74.0	13.50 FPU/g of *Trichoderma reesei* ATCC 26921 and 16.50 U/g *β*-glucosidase enzyme by *Almonds Lyophyl*	[46]

Corn cob	Alkali pretreatment	438.47		717.0 U/g of xylanase, 77 U/gds of CMCase, and 26.12 U/gds of FPase by *Aspergillus candidus*	[40]

Wheat straw Rice straw Corncobs	Alkali pretreatment	193.86 178.93 171.06		2400 U/g of xylanase from *Aspergillus foetidus* MTCC 4898	[41]

Apple pomace	Acid pretreatment and polyphenol degradation	266.0	—	43.0 U/g of Celluclast 1.5 L, 183.0 U/g of Pectinex 3XL, and 41.0 U/g of Novozyme 188 (Novozymes)	[44]

*Cistus ladanifer* *Cytisus striatus*	Acid pretreatment	313.0 448.0	—	60.0 FPU/g of Cellulase complex NS50013 and *∗* U/g *β*-glucosidase NS50010 (Novozymes)	[45]

Oil palm empty fruit bunches	Alkali pretreatment	534.53		10% (v/v) of crude cellulase enzyme by *Trichoderma reesei* RUT C-30	[47]

*Populus nigra*	Alkali pretreatment	667.0		25 FPU/g of cellulase by *Pholiota adiposa* SKU0714	[48]

*Parthenium* sp.	Alkali pretreatment	513.0		7 FPU/g of Accellerase 1500 (Genencor)	[49]

Sweet sorghum straw	Acid pretreatment	440.0	—	25 FPU/g of cellulase by *Coriolus versicolor* TD17	[43]

*Gracilaria* sp.	Acid pretreatment	592.0		0.01 g/g of commercial cellulase	[50]

Paddy straw or *Parthenium* sp.	Biological pretreatement by *Myrotechium roridum* LG7	509.65		*∗* U/g of Accellerase 1500 (Novozymes)	[49]

*Arundo donax*	Acid pretreatment	264.0	217.0	60 FPU/g cellulose of cellulases and 64 pNPGU/g cellulose *β*-glucosidases	[38]

*Parthenium* sp.	Biological pretreatement by *Trametes hirsuta*	485.64		*∗* U/g of Accellerase 1500 (Novozymes)	[39]

Corn stover	Acid pretreatment	545.0		*∗* U/g of Accellerase 1500 and *∗* U/g of Accellerase XY (Genencor)	[51]

*Populus balsamifera*	Alkali pretreatments	293.0		100 FPU/g of cellulase by *Agaricus arvensis*	[42]

*Arundo donax*	Steam explosion	229.3	250.8	20 U/gds and 400 U/gds of cellulase and xylanase, respectively, from *Pleurotus ostreatus*, supplemented with the commercial enzymatic mix C	This study
		480.10			

^*∗*^In the paper the enzymatic units were not reported.
