# Supplementary material for: Optimization of Arundo donax Saccharification by (Hemi)cellulolytic Enzymes from Pleurotus ostreatus
Source: Biomed Res Int. 2015 Nov 5;2015:951871. doi: 10.1155/2015/951871 (PMC4652331; doi:10.1155/2015/951871)
Supplement: Supplementary file 1 — In the supplementary materials are provided: Table S1 indicating the levels decoded of the tested parameters in the Plackett-Burman Screening Design; Table S2 in which are reported the independent factors (Temperature, Time and pH) and their respective coded and decoded levels used for the 33 experimental design of the optimization of the enzymatic hydrolysis of Arundo donax; Table S3 showing the Analysis of Variance (Anova) of Plackett-Burman Screening Design for the enzymatic hydrolysis of Arundo donax; Table S4 showing the Anova of the 33 factorial experimental design for the optimization of enzymatic hydrolysis parameters and Table S5 in which is reported the comparison of observed and predicted values of the 33 factorial experimental design of sugars released (mg/gds) during the enzymatic hydrolysis of Arundo donax. Moreover, they provided: Figure S1 showing the inducer effect of 1% microcrystalline cellulose on cellulase and xylanase activities production by Pleurotus ostreatus in 24-Multiwell; Figure S2 showing the inductive effect of 1% microcrystalline cellulose on cellulase and xylanase activities production by Pleurotus ostreatus in 1 L Erlenmeyer flask and Figure S3 in which is reported the correlation between observed and predicted values of the total sugars released by enzymatic hydrolysis of Arundo donax. [file 951871.f1.doc]

**Supplemetal data**

**Tables**

**Tables S1.** Levels decoded of the tested parameters in the Plackett-Burman Screening Design.

|  | **Levels** | | |
| --- | --- | --- | --- |
| **Variables** | **-1** | **0** | **+1** |
| Temperature (°C) | 25 | 35 | 45 |
| % Biomass (w/v) | 3 | 5 | 7 |
| pH | 3 | 4.5 | 6 |
| Cellulase from PO  (U/gds) | 10 | 20 | 30 |
| Cellulase from *Trichoderma reesei* ATCC26921 (U/gds) | 1.8 | 3.6 | 5.4 |
| Time (hours) | 24 | 48 | 72 |

**Tables S2.** Independent factors (Temperature, Time and pH) and their respective coded and decoded levels used for the 3^3^ experimental design of the optimization of the enzymatic hydrolysis of *Arundo donax*.

|  | **Levels** | | |
| --- | --- | --- | --- |
| **Variables** | **-1** | **0** | **+1** |
| Temperature (°C) | 35 | 45 | 55 |
| Time (hours) | 48 | 72 | 96 |
| pH | 2.5 | 3 | 3.5 |

**Tables S3**. Analysis of Variance (Anova) of Plackett-Burman Screening Design for the enzymatic hydrolysis of *Arundo donax*.

|  |  | **SS**^a^ | **df**^b^ | **MS**^c^ | **F** | ***p*** |
| --- | --- | --- | --- | --- | --- | --- |
| **R^2^ = 0.98; R^2^Adj = 0.92^d^** | | | | | | |
|  | **(A) Temperature (°C)^e^** | **32881.0** | **1** | **32881.0** | **22.14741** | **0.018157** |
|  | (B) % biomass (w/v) | 5609.5 | 1 | 5609.5 | 3.77836 | 0.147163 |
|  | **(C) pH^e^** | **121313.6** | **1** | **121313.6** | **81.71218** | **0.002859** |
|  | (D) Cellulase from PO  (U/gds) | 6728.4 | 1 | 6728.4 | 4.53202 | 0.123145 |
|  | (E) Cellulase from  *Trichoderma reesei*  ATCC26921 (U/gds) | 12828.2 | 1 | 12828.2 | 8.64059 | 0.060535 |
|  | **(F) Time (hours)^e^** | **15534.0** | **1** | **15534.0** | **10.46311** | **0.048045** |
|  | (G) Dummy 1 | 6.2 | 1 | 6.2 | 0.00420 | 0.952405 |
|  | (H) Dummy 2 | 5661.5 | 1 | 5661.5 | 3.81339 | 0.145875 |
|  | (I) Dummy 3 | 3265.4 | 1 | 3265.4 | 2.19941 | 0.234694 |
|  | **(L) Dummy 4^e^** | **42548.5** | **1** | **42548.5** | **28.65902** | **0.012751** |
|  | (M) Dummy 5 | 76.8 | 1 | 76.8 | 0.05170 | 0.834741 |
|  | Error | 4453.9 | 3 | 1484.6 |  |  |
|  | Total SS | 250907.1 | 14 |  |  |  |

^a^ SS: Sum of Squares.

^b^ df: Degrees of freedom.

^c^ MS: Mean Square.

^d^ R^2^: coefficient of determination and R^2^ _adj_: coefficient of determination adjusted.

^e^ Significant factors/variables at p<0.05.

**Table S4.** Analysis of Variance (Anova) of the 3^3^ factorial experimental design for the optimization of enzymatic hydrolysis parameters.

|  |  | **SS**^a^ | **df**^b^ | **MS**^c^ | **F** | **P** |
| --- | --- | --- | --- | --- | --- | --- |
| **R^2^ = 0.93; R^2^_Adj_ = 0.80** | | | | | | |
|  | **(A) Temperature (°C)^e^ L+Q** | **35420.3** | **2** | **17710.16** | **14.10352** | **0.000921** |
|  | **(B) Time (hours)^e^ L+Q** | **40494.4** | **2** | **20247.21** | **16.12391** | **0.000537** |
|  | **(C) pH L+Q** | **52205.0** | **2** | **26102.50** | **20.78679** | **0.000183** |
|  | **A by B^e^** | **34855.5** | **4** | **8713.86** | **6.93931** | **0.004846** |
|  | A by C | 6108.9 | 4 | 1527.23 | 1.21621 | 0.358472 |
|  | B by C | 2514.5 | 4 | 628.63 | 0.50061 | 0.736200 |
|  | Error | 13813.0 | 11 | 1255.73 |  |  |
|  | Total SS | 185756.1 | 29 |  |  |  |

^a^ SS: Sum of Squares.

^b^ df: Degrees of freedom.

^c^ MS: Mean Square.

^d^ R^2^: coefficient of determination and R^2^ _adj_: coefficient of determination adjusted.

^e^ Significant factors/variables at p<0.05.

**Table S5.** Comparison of observed and predicted values of the 3^3^ factorial experimental design of sugars released (mg/gds) during the enzymatic hydrolysis of *Arundo donax*.

|  | | | | **Total sugars released**  **(mg/gds)** | | |  |
| --- | --- | --- | --- | --- | --- | --- | --- |
| **Run** | **Temperature (°C)** | **Time (hours)** | **pH** | **Observed value** | **Predicted value** | |  |
| **1** | 35 | 48 | 2,5 | 289.90 | 268.99 |  | |
| **2** | 35 | 48 | 3 | 264.40 | 272.29 |  | |
| **3** | 35 | 48 | 3,5 | 353.30 | 366.32 |  | |
| **4** | 35 | 72 | 2,5 | 184.60 | 196.88 |  | |
| **5** | 35 | 72 | 3 | 180.60 | 187.93 |  | |
| **6** | 35 | 72 | 3,5 | 315.80 | 296.18 |  | |
| **7** | 35 | 96 | 2,5 | 187.50 | 196.13 |  | |
| **8** | 35 | 96 | 3 | 233.80 | 218.57 |  | |
| **9** | 35 | 96 | 3,5 | 335.90 | 342.50 |  | |
| **10** | 45 | 48 | 2,5 | 196.90 | 163.82 |  | |
| **11** | 45 | 48 | 3 | 209.10 | 207.37 |  | |
| **12** | 45 | 48 | 3,5 | 233.90 | 268.71 |  | |
| **12** | 45 | 72 | 2,5 | 150.30 | 174.96 |  | |
| **14** | 45 | 72 | 3 | 207.40 | 206.25 |  | |
| **15** | 45 | 72 | 3,5 | 291.70 | 281.82 |  | |
| **16** | 45 | 96 | 2,5 | 292.80 | 301.22 |  | |
| **17** | 45 | 96 | 3 | 347.40 | 363.91 |  | |
| **18** | 45 | 96 | 3,5 | 480.10 | 455.16 |  | |
| **19** | 55 | 48 | 2,5 | 69.40 | 123.39 |  | |
| **20** | 55 | 48 | 3 | 185.0 | 178.84 |  | |
| **21** | 55 | 48 | 3,5 | 235.60 | 187.76 |  | |
| **22** | 55 | 72 | 2,5 | 156.70 | 119.76 |  | |
| **23** | 55 | 72 | 3 | 155.50 | 162.95 |  | |
| **24** | 55 | 72 | 3,5 | 156.60 | 186.09 |  | |
| **25** | 55 | 96 | 2,5 | 203.18 | 186.12 |  | |
| **26** | 55 | 96 | 3 | 262.0 | 260.71 |  | |
| **27** | 55 | 96 | 3,5 | 281.0 | 299.54 |  | |
| **28C** | 45 | 72 | 3 | 210.9 | 206.25 |  | |
| **29C** | 45 | 72 | 3 | 211.0 | 206.25 |  | |
| **30C** | 45 | 72 | 3 | 210.5 | 206.25 |  | |

**Supplementary Figures**

**Figure S1.** Inducer effect of 1% microcrystalline cellulose on **A)** cellulase and **B)** xylanase activities production by *Pleurotus ostreatus* in 24-Multiwell.

**Figure S2.** Inductive effect of 1% microcrystalline cellulose on **A)** cellulase and **B)** xylanase activities production by *Pleurotus ostreatus* in 1 L Erlenmeyer flask.

**Figure S3.** Correlation between observed and predicted values of the total sugars released by enzymatic hydrolysis of *Arundo donax*.
